# Occupational Class Inequalities in All-Cause and Cause-Specific Mortality among Middle-Aged Men in 14 European Populations during the Early 2000s

**DOI:** 10.1371/journal.pone.0108072

**Published:** 2014-09-30

**Authors:** Marlen Toch-Marquardt, Gwenn Menvielle, Terje A. Eikemo, Ivana Kulhánová, Margarete C. Kulik, Matthias Bopp, Santiago Esnaola, Domantas Jasilionis, Netta Mäki, Pekka Martikainen, Enrique Regidor, Olle Lundberg, Johan P. Mackenbach, for the Euro-GBD-SE consortium

**Affiliations:** 1 Department of Sociology and Political Science, NTNU, Trondheim, Norway; 2 Department of Public Health, Erasmus MC, Rotterdam, the Netherlands; 3 INSERM, UMR_S 1136, Pierre Louis Institute of Epidemiology and Public Health, Paris, France; 4 Sorbonne Universités, UPMC Univ Paris 06, UMR_S 1136, Pierre Louis Institute of Epidemiology and Public Health, Paris, France; 5 Institute of Social and Preventive Medicine, University of Zurich, Zurich, Switzerland; 6 Health Studies and Research Unit, Department of Health and Consumer Affairs, Basque Government, Donostia-San Sebastián 1, Vitoria-Gasteiz, Spain; 7 Max-Planck-Institute for Demographic Research, Rostock, Germany; 8 Population Research Unit, Department of Sociology, University of Helsinki, Helsinki, Finland; 9 Department of Preventive Medicine and Public Health, School of Medicine, Universidad Complutense de Madrid, Ciudad Universitaria, 28040 Madrid, Spain; 10 CHESS, Stockholm University/Karolinksa Institutet, Stockholm, Sweden; Geisel School of Medicine at Dartmouth College, United States of America

## Abstract

This study analyses occupational class inequalities in all-cause mortality and four specific causes of death among men, in Europe in the early 2000s, and is the most extensive comparative analysis of occupational class inequalities in mortality in Europe so far. Longitudinal data, obtained from population censuses and mortality registries in 14 European populations, from around the period 2000–2005, were used. Analyses concerned men aged 30–59 years and included all-cause mortality and mortality from all cancers, all cardiovascular diseases (CVD), all external, and all other causes. Occupational class was analysed according to five categories: upper and lower non-manual workers, skilled and unskilled manual workers, and farmers and self-employed combined. Inequalities were quantified with mortality rate ratios, rate differences, and population attributable fractions (PAF). Relative and absolute inequalities in all-cause mortality were more pronounced in Finland, Denmark, France, and Lithuania than in other populations, and the same countries (except France) also had the highest PAF values for all-cause mortality. The main contributing causes to these larger inequalities differed strongly between countries (e.g., cancer in France, all other causes in Denmark). Relative and absolute inequalities in CVD mortality were markedly lower in Southern European populations. We conclude that relative and absolute occupational class differences in all-cause and cause specific mortality have persisted into the early 2000's, although the magnitude differs strongly between populations. Comparisons with previous studies suggest that the relative gap in mortality between occupational classes has further widened in some Northern and Western European populations.

## Introduction

In the last decades life expectancy in industrialised countries has shown a remarkable increase [1], [2]. Substantial differences in mortality between socioeconomic groups, however, are still observed and might even be increasing in Europe [3]–[5]. These inequalities are one of the most important challenges of public health [6]. Cross-country comparisons can help identify the scope for reduction. Europe is a unique region in this respect, as the magnitude of inequalities in health has been shown to differ substantially between countries [7].

Most previous comparative research documented educational differences in mortality. Only a few studies have analysed occupational class differences in mortality across Europe. These mostly focused on the 1970s, 1980s, and 1990s, and not always took economically inactive persons into account, due to lacking information on their occupational class [8]–[10]. Inactive persons generally have a higher mortality when compared with active persons from the same occupational class, and more often have a manual occupation [11]. Therefore, disregarding them in mortality analyses by occupational class can lead to an underestimation of mortality differences [11].

Because of the different characteristics of education and occupational class, both measures cannot be used interchangeably when studying socioeconomic inequalities in mortality [12]. While education is attained at younger ages and remains more or less fixed in adult life, occupational class can change throughout the life course [13]. Education reflects knowledge attainment, intellectual resources, and cognitive functioning, and may also reflect the ability to take up health education and health innovations [14]. Occupational class is more directly related to income and material resources, and thus reflects good access to health care, social positioning, social networks, as well as work-related factors such as stress, control, autonomy, and occupational hazards [14], [15]. Levels of education and occupation are usually correlated, because education influences the occupational class acquired. Furthermore, while education captures the human capital acquired earlier in life, occupational class reflects the possibility to realise that human capital in the labour market. In view of these differences between education and occupational class there is thus a clear need to analyse both in relation to health outcomes. However, in this study we are focusing on occupational class, as most international comparative studies of health inequalities use education as indicator of socioeconomic position [16]–[18].

The aim of this study is to analyse occupational class inequalities in all-cause and cause-specific mortality among men, in 14 European populations in the early 2000s. In this study occupational class was operationalized with the Erikson-Goldthorpe (EGP) social class scheme [19]. Because the availability of mortality data by occupational class has increased since the 1990s, this is not only the most recent, but also the most extensive comparative analysis of occupational class inequalities in mortality in Europe ever conducted.

## Data and Methods

Ethics approval was not required for this paper because it consists of unidentifiable data.

Data cannot be made publicly available, but an overview of persons and institutions who provided data to the study can be found in Eikemo and Mackenbach [20]. A written informed consent of the usage of mortality and National Health Interview Survey data was given by the relevant administrative units from all participating countries.

We used longitudinal data obtained from population censuses and mortality registries from 14 European populations, from around the period 2000–2005. For detailed characteristics of the data see Table 1. Data were centrally harmonized to enhance cross-country comparability and contained information on sex, age, occupational class, employment status, and cause-specific mortality. The populations ranged from Finland, Denmark, and Sweden in the North, to England and Wales, Scotland, Netherlands, France, Switzerland and Austria in the West, Spain (represented by the Basque Country and the region of Madrid) and Italy (represented by the cities of Turin and of Florence, Leghorn and Prato (Tuscany)) in the South, and Lithuania in the Baltic region. Because some countries (Denmark, Sweden, and Switzerland) used age-at-baseline for the classification of deaths, instead of age-at-death, a correction factor taking into account the length of follow-up was developed and validated [21], and used in the analyses to obtain comparable ages-at-death for these countries.

**Table 1:** Characteristics of the mortality data used, men, age 30–59.

Population	Type of dataset	Period	Geographic coverage	Demographic coverage	Person-years of follow-up	Number of deaths
NORTH						
**Finland**	longitudinal	2001–2007	National	20% of Finns are excluded (at random)	5,742,810	18,216
**Sweden**	longitudinal	2001–2006	National	whole population	11,000,000	18,894
**Denmark**	longitudinal	2001–2005	National	whole population	5,708,058	14,837
WEST						
**England & Wales**	longitudinal	2001–2006	National	1% of the population	516,971	976
**Scotland**	longitudinal	2001–2006	National	whole population		472
**Netherlands**	longitudinal	1998–2003/2003–2007	National	from labour force survey	708,457	1,154
**France**	longitudinal	1999–2005	National	1% of the population (born outside France mainland excluded)	579,691	1,428
**Switzerland**	longitudinal	2001–2005	National	Non-Swiss nationals excluded	6,091,605	10,220
**Austria**	longitudinal	2001–2002	National	whole population	1,747,710	3,638
SOUTH						
**Spain (Basque)**	longitudinal	2001–2006	Regional	whole population	2,359,033	5,518
**Spain (Madrid)**	cross-sectional, linked	2001–2003	Regional	whole population	1,902,232	3,691
**Italy (Turin)**	longitudinal	2001–2006	Urban	whole population	784,020	1,310
**Italy (Tuscany)**	longitudinal	2001–2005	Florence, Leghorn, Prato	whole population	473,682	699
BALTIC						
**Lithuania**	longitudinal	2001–2005	National	whole population	3,149,964	28,532

Causes of death were coded according to the International Classification of Diseases (ICD)-10 and comprised all-cause mortality, mortality from all cancers (C00-D48), all cardiovascular diseases (CVD, I00-I99), all external causes (V01-Y98), and all other causes (remaining ICD codes).

Occupational class was classified following the EGP scheme which was initially developed for international comparisons and has already been used in several studies on occupational class inequalities in mortality[5], [22]. We analysed occupational class in five categories: upper non-manual workers (professionals, managers), lower non-manual workers (clerical, service, sales workers), skilled manual workers, unskilled manual workers, and farmers and self-employed combined. This detailed classification of occupation was not available in Switzerland (non-manual workers combined and manual workers combined) and in Austria (non-manual workers were combined).

Men aged 30 to 59 were analysed. Women were excluded from the analysis, as in most countries information on occupational class was missing for more than 30% of women. Information on occupational class was lacking for economically inactive men (between 9% in Austria and 29% in Lithuania) in most datasets. We therefore applied a correction algorithm, developed by Kunst and Groenhof [23], to account for inactivity when computing occupational class differences in mortality. Three types of input information were necessary for applying the algorithm: the relative difference of the proportion of inactive men by occupational class when compared with the total population; the proportion of inactive men in the total population; and the mortality rate ratio of inactive versus active men. The first input was obtained from National Health Interview Surveys, conducted during the late 1990s and the early 2000s, whereas the two latter inputs were calculated directly from the mortality datasets. The algorithm was applied separately for each population and cause of death, and is presented in more detail in Appendix S1. We evaluated the performance of the algorithm in four populations where information on occupational class was available for inactive men: Finland, England and Wales, Basque Country, and Turin (see Appendix S2). The analyses show that the correction works well and adequately corrects for the exclusion of inactive men. For the sake of consistency, the results for all countries as presented in this paper were obtained using the correction procedure.

Age-standardised mortality rates were computed per 100,000 person-years of exposure and directly standardised with the European standard population [24]. In order to assess the magnitude of occupational class inequalities, rate ratios adjusted for age with 95% confidence intervals (CI) were estimated with Poisson regression, using upper non-manual workers as reference category. Absolute inequalities were expressed as age-standardized mortality rate differences between unskilled manual and upper non-manual workers. We also calculated the population attributable fraction (PAF), measuring the share of deaths that might be avoided if all occupational groups experienced the mortality of the upper non-manual workers. It is calculated as follows:
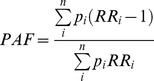
(1)where RR_i_ is the mortality rate ratio and p_i_ is the proportion of the population in occupational class i. All analyses were conducted using Stata, version 12.

## Results

Large differences in the occupational class distribution were found between populations (Table 2). The highest shares of upper non-manual workers were found in Western Europe and Tuscany where they ranged from 40% to 50%. The highest shares of unskilled manual workers were found in Denmark, Sweden, England and Wales, Scotland, and the Basque Country (between 20% and 25%).

**Table 2:** Distribution of economically active men (%), age-standardised mortality rate (ASMR)^a^ and rate ratio (RR) of all-cause and cause-specific mortality by occupational class, men, age 30–59^b^.

		All causes			All cancer			All CVD			All external			All other		
	%	ASMR	RR	(95% CI)	ASMR	RR	(95% CI)	ASMR	RR	(95% CI)	ASMR	RR	(95% CI)	ASMR	RR	(95% CI)
NORTH																
**Finland**																
Upper non-manual	19	187	1		50	1		50	1		53	1		34	1	
Lower non-manual	20,4	295	1,57	(1.48–1.66)	68	1,39	(1.24–1.55)	83	1,64	(1.47–1.83)	79	1,47	(1.32–1.65)	64	1,87	(1,64–2,13)
Skilled manual	26,5	454	2,4	(2.28–2.53)	84	1,7	(1.54–1.89)	120	2,34	(2.12–2.58)	143	2,66	(2.42–2.94)	107	3,09	(2.75–3.48)
Unskilled manual	20,3	645	3,42	(3.24–3.60)	103	2,09	(1.88–2.32)	176	3,46	(3.37–3.64)	204	3,78	(3.43–4.17)	163	4,75	(4.23–5.34)
Farmers & self-employed	13,8	272	1,42	(1.33–1.50)	54	1,1	(0.97–1.24)	79	1,52	(1.53–1.70)	89	1,6	(1.43–1.80)	50	1,44	(1.25–1.66)
**Sweden**																
Upper non-manual	30,8	151	1		51	1		34	1		30	1		15	1	
Lower non-manual	9,3	237	1,57	(1.49–1.66)	62	1,23	(1.11–1.36)	54	1,58	(1.41–1.77)	39	1,34	(1.17–1.54)	35	2,4	(2.05–2.82)
Skilled manual	23,8	289	1,92	(1.84–2.00)	71	1,41	(1.30–1.52)	61	1,77	(1.62–1.94)	63	2,14	(1.94–2.35)	44	2,97	(2.62–3.37)
Unskilled manual	29,7	376	2,49	(2.39–2.59)	79	1,56	(1.45–168)	83	2,42	(2.23–2.63)	71	2,4	(2.19–2.63)	64	4,34	(3.86–4.88)
Farmers & self-employed	6,4	205	1,34	(1.27–1.42)	63	1,2	(1.08–1.33)	43	1,3	(1.15–147)	46	1,54	(1.34–1.77)	24	1,52	(1.27–1.82)
**Denmark**																
Upper non-manual	18,4	157	1		69	1		33	1		19	1		32	1	
Lower non-manual	12,5	234	1,45	(1.33–1.57)	79	1,14	(1.01–1.29)	53	1,58	(1.34–1.86)	32	1,59	(1.29–1.97)	54	1,66	(1.40–1.97)
Skilled manual	36,4	366	2,36	(2.22–2.52)	110	1,58	(1.45–1.73)	72	2,18	(1.92–2.47)	58	3,03	(2.58–3.57)	99	3,12	(2.75–3.54)
Unskilled manual	20,5	526	3,47	(3.25–3.70)	138	1,99	(1.81–2.19)	95	2,84	(2.49–3.24)	77	3,99	(3.37–4.72)	164	5,12	(4.50–5.83)
Farmers & self-employed	12,2	230	1,47	(1.36–1.58)	84	1,22	(1.09–1.36)	47	1,4	(1.20–1.64)	43	2,19	(1.81–2.65)	54	1,69	(1.45–1.98)
WEST																
**England & Wales**																
Upper non-manual	42,7	174	1		77	1		49	1		24	1		24	1	
Lower non-manual	5,5	183	1,05	(0.76–1.47)	59	0,77	(0.44–1.36)	55	1,12	(0.61–2.04)	12	0,59	(0.18–1.90)	58	2,31	(1.20–4.44)
Skilled manual	13,5	278	1,59	(1.30–1.94)	101	1,31	(0.96–1.79)	90	1,78	(1.25–2.54)	27	1,21	(0.66–2.20)	58	2,36	(1.45–3.83)
Unskilled manual	22,9	371	2,12	(1.81–2.48)	92	1,18	(0.90–1.54)	125	2,51	(1.90–3.31)	57	2,44	(1.60–3.71)	99	4,13	(2.84–5.99)
Farmers & self-employed	15,4	229	1,28	(1.06–1.54)	80	1	(0.75–1.35)	72	1,4	(1.01–1.96)	42	1,82	(1.13–2.95)	35	1,44	(0.89–2.32)
**Scotland**																
Upper non-manual	39,6	146	1		60	1		43	1		15	1		28	1	
Lower non-manual	6,3	192	1,4	(1.16–2.30)	69	1,11	(0.53–2.33)	34	0,8	(0.29–2.22)	42	3,41	(1.42–8.71)	43	1,65	(0.54–3.64)
Skilled manual	15,7	249	1,72	(1.47–3.08)	74	1,23	(0.77–1.97)	63	1,44	(0.84–2.46)	41	2,78	(1.37–6.18)	65	2,5	(1.06–3.56)
Unskilled manual	25,6	398	2,7	(2.40–4.63)	111	1,85	(1.29–2.66)	105	2,37	(1.57–3.58)	65	4,08	(2.29–8.26)	103	3,91	(1.69–4.58)
Farmers & self-employed	12,8	175	1,25	(0.93–1.69)	61	1,04	(0.64–1.70)	58	1,32	(0.77–2.24)	23	1,83	(0.77–4.38)	33	1,26	(0.61–2.48)
**Netherlands**																
Upper non-manual	43,5	160	1		81	1		41	1		22	1		26	1	
Lower non-manual	10,8	210	1,33	(0.91–2.16)	94	1,17	(0.86–1.60)	68	1,71	(1.20–2.44)	26	1,25	(0.70–2.22)	28	1,18	(0.69–2.00)
Skilled manual	14,6	239	1,5	(1.30–2.28)	94	1,16	(0.88–1.54)	76	1,91	(1.40–2.61)	37	1,61	(1.00–2.59)	37	1,66	(1.08–2.56)
Unskilled manual	14,9	318	2,04	(2.16–3.37)	125	1,6	(1.25–2.06)	92	2,3	(1.71–3.10)	49	2,4	(1.57–3.67)	52	2,45	(1.66–3.62)
Farmers & self-employed	16,2	174	1,11	(0.94–1.32)	73	0,92	(0.70–1.21)	52	1,31	(0.95–1.80)	20	1	(0.59–1.69)	37	1,43	(0.97–2.12)
**France**																
Upper non-manual	39,9	217	1		98	1		33	1		43	1		42	1	
Lower non-manual	8,2	479	2,29	(1.91–2.74)	184	1,88	(1.42–2.50)	47	1,67	(0.98–2.86)	110	2,64	(1.83–3.81)	135	3,23	(2.23–4.68)
Skilled manual	25	443	2,02	(1.76–2.31)	178	1,76	(1.44–2.16)	78	2,47	(1.76–3.47)	76	1,79	(1.33–2.42)	107	2,4	(1.78–3.25)
Unskilled manual	13,8	598	2,73	(2.34–3.19)	234	2,3	(1.81–2.92)	81	2,62	(1.72–3.97)	115	2,65	(1.92–3.67)	158	3,66	(2.61–5.13)
Farmers & self-employed	13,1	235	1,08	(0.90–1.29)	81	0,79	(0.59–1.06)	41	1,33	(0.85–2.06)	56	1,32	(0.90–1.94)	57	1,34	(0.92–1.97)
**Switzerland** c ^,^ d																
Upper non-manual	57,1	162	1		59	1		36	1		35	1		32	1	
Lower non-manual	-	-	-	-	-	-	-	-	-	-	-	-	-	-	-	-
Skilled manual	17,6	320	2,15	(2.04–2.26)	105	1,77	(1.62–1.94)	64	1,76	(1.57–1.98)	65	1,85	(1.66–2.07)	74	2,32	(2.07–2.61)
Unskilled manual	-	-	-	-	-	-	-	-	-	-	-	-	-	-	-	-
Farmers & self-employed	25,3	180	1,13	(1.07–1.19)	61	1,03	(0.94–1.12)	41	1,12	(1.00–1.24)	50	1,42	(1.28–1.57)	27	0,86	(0.76–0.97)
**Austria** c																
Upper non-manual	48,2	199	1		67	1		60	1		48	1		24	1	
Lower non-manual	-	-	-	-	-	-	-	-	-	-	-	-	-	-	-	-
Skilled manual	32,2	317	1,34	(1.23–1.46)	90	1,13	(0.96–1.32)	85	1,19	(1.02–1.40)	84	1,58	(1.36–1.84)	53	1,69	(1.35–2.11)
Unskilled manual	6,4	400	1,74	(1.51–2.02)	151	1,78	(1.37–2.31)	106	1,71	(1.31–2.25)	61	1,29	(0.95–1.76)	77	2,65	(1.83–3.86)
Farmers & self-employed	13,2	196	1,42	(1.27–1.59)	58	1,17	(0.95–1.45)	59	1,26	(1.02–1.56)	63	1,68	(1.38–2.05)	18	1,43	(1.01–2.03)
SOUTH																
**Spain (Basque)**																
Upper non-manual	24	184	1		98	1		38	1		20	1		27	1	
Lower non-manual	14,9	280	1,53	(1.39–1.69)	137	1,41	(1.23–1.62)	62	1,6	(1.29–1.97)	34	1,67	(1.27–2.19)	45	1,71	(1.33–2.20)
Skilled manual	36,6	335	1,86	(1.72–2.01)	150	1,54	(1.38–1.72)	68	1,79	(1.51–2.13)	53	2,64	(2.13–3.28)	62	2,39	(1.95–2.93)
Unskilled manual	21,7	412	2,29	(2.11–2.49)	181	1,86	(1.65–2.10)	78	2,06	(1.71–2.49)	55	2,74	(2.18–3.46)	99	3,82	(3.10–4.70)
Farmers & self-employed	2,8	325	1,8	(1.54–2.11)	135	1,4	(1.10–1.78)	66	1,73	(1.22–2.45)	53	2,19	(1.81–2.65)	70	1,69	(1.45–1.98)
**Spain (Madrid)**																
Upper non-manual	26,9	202	1		97	1		39	1		19	1		47	1	
Lower non-manual	34,7	297	1,51	(1.38–1.65)	117	1,21	(1.06–1.39)	63	1,65	(1.35–2.02)	29	1,61	(1.23–2.11)	86	1,9	(1.60–2.27)
Skilled manual	30,3	339	1,71	(1.57–1.88)	146	1,5	(1.32–1.72)	55	1,45	(1.17–1.79)	39	2,14	(1.64–2.80)	96	2,11	(1.77–2.52)
Unskilled manual	7,2	458	2,3	(2.03–2.61)	188	1,95	(1.62–2.36)	69	1,83	(1.35–2.49)	39	1,94	(1.30–2.90)	164	3,53	(2.80–4.46)
Farmers & self-employed	0,9	326	1,68	(1.24–2.26)	142	1,47	(0.92–2.33)	74	1,96	(1.03–3.73)	29	1,84	(0.75–4.55)	81	1,77	(0.97–3.23)
**Italy (Turin)**																
Upper non-manual	24,7	158	1		68	1		48	1		23	1		19	1	
Lower non-manual	20,2	219	1,4	(1.12–1.59)	99	1,46	(1.08–1.83)	53	1,17	(0.82–1.59)	23	0,97	(0.57–1.54)	42	2,25	(1.25–3.18)
Skilled manual	18,5	288	1,81	(1.44–2.04)	127	1,87	(1.39–2.33)	69	1,47	(1.02–1.98)	30	1,17	(0.69–1.84)	61	3,18	(1.76–4.40)
Unskilled manual	17,8	346	2,21	(1.84–2.56)	144	2,07	(1.59–2.64)	66	1,47	(1.05–2.02)	39	1,68	(1.06–2.61)	103	5,49	(3.39–7.86)
Farmers & self-employed	18,8	218	1,39	(1.17–1.65)	79	1,16	(0.88–1.52)	61	1,33	(0.97–1.82)	39	1,65	(1.07–2.54)	40	2,04	(1.29–3.24)
**Italy (Tuscany)**																
Upper non-manual	47,5	153	1		72	1		36	1		20	1		25	1	
Lower non-manual	15,6	202	1,31	(1.05–1.63)	102	1,43	(1.05–1.95)	35	0,96	(0.58–1.59)	22	0,95	(0.50–1.81)	42	1,78	(1.07–2.97)
Skilled manual	26,4	236	1,56	(1.30–1.86)	98	1,38	(1.06–1.80)	45	1,27	(0.86–1.87)	36	1,66	(1.05–2.62)	57	2,4	(1.58–3.64)
Unskilled manual	10,1	263	1,72	(1.35–2.20)	108	1,49	(1.03–2.16)	51	1,43	(0.83–2.45)	35	1,61	(0.84–3.06)	69	2,98	(1.71–5.18)
Farmers & self-employed	0,4	299	1,95	(0.81–4.73)	48	0,82	(0.11–5.85)	250	6,31	(2.30–17.26)	0	0		0	0	
BALTIC																
**Lithuania**																
Upper non-manual	13,7	317	1		75	1		110	1		105	1		28	1	
Lower non-manual	2	505	1,55	(1.33–1.79)	131	1,77	(1.30–2.39)	163	1,49	(1.14–1.94)	129	1,24	(0.96–1.59)	82	2,87	(1.93–4.24)
Skilled manual	25,1	717	2,24	(2.12–2.41)	148	1,93	(1.69–2.23)	221	1,98	(1.78–2.23)	249	2,34	(2.12–2.62)	92	3,34	(2.75–4.17)
Unskilled manual	23,1	826	2,56	(2.43–2.77)	158	2	(1.76–2.34)	245	2,18	(1.97–2.49)	306	2,85	(2.60–3.21)	103	3,73	(3.08–4.72)
Farmers & self-employed	36,1	648	2,86	(2.69–3.04)	113	2,04	(1.78–2.34)	172	2,17	(1.95–2.42)	275	3,59	(3.26–3.97)	89	4,53	(3.73–5.51)

a Direct age standardised mortality rates (deaths per 100,000 person-years).

b Results are corrected for exclusion of economically inactive.

c Both non-manual categories combined.

d Both manual categories combined.

Table 2 shows age-standardised mortality rates and mortality rate ratios for all-cause and cause-specific mortality for five occupational groups. Mortality rates were higher among manual than among non-manual workers in all populations and for all causes of death. All-cause-mortality rates were particularly high in Lithuania, where non-manual workers even had higher mortality rates than manual workers in most other countries.

**Figure 1:**
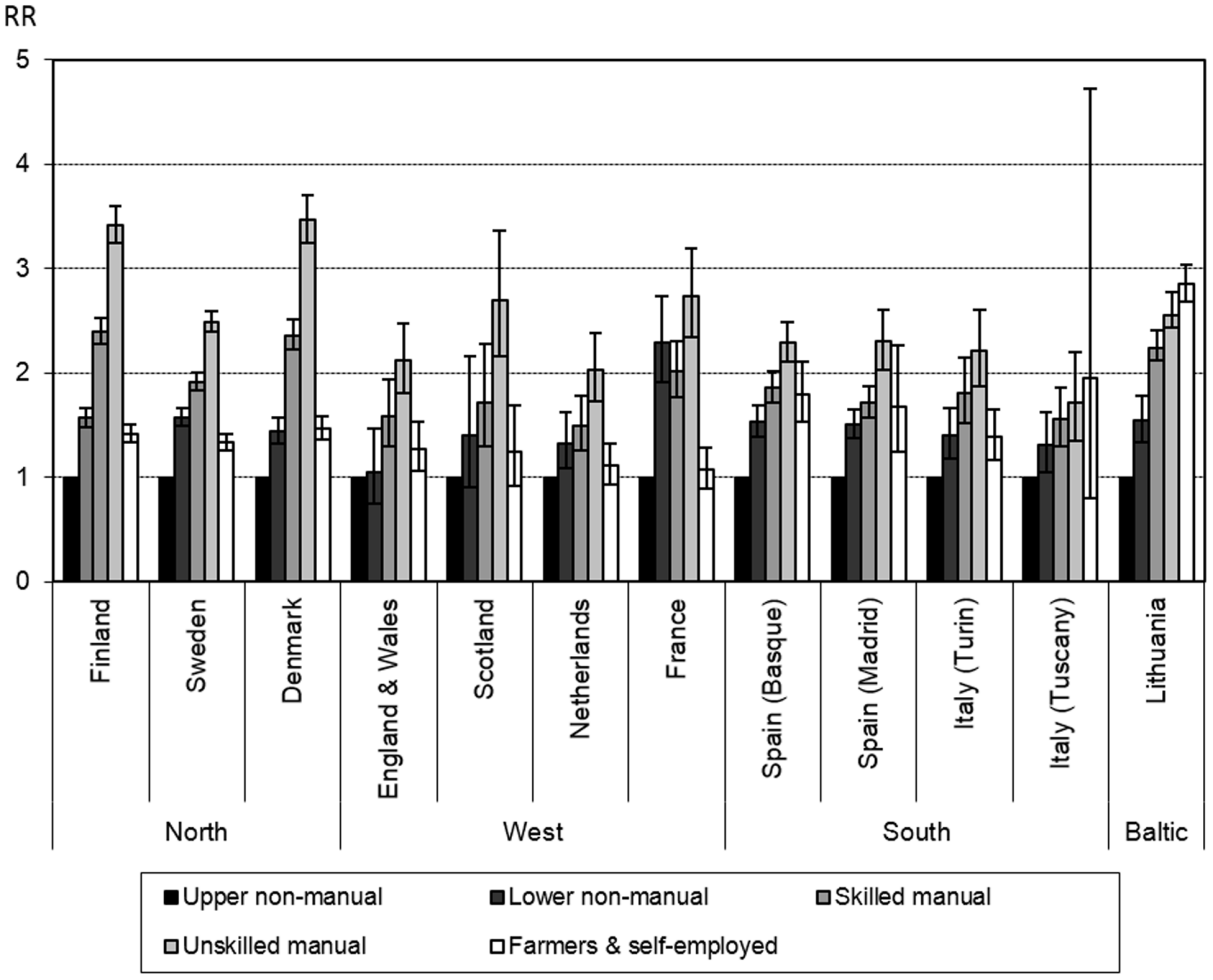
Rate ratio of all-cause mortality by occupational class, men, age 30–59.^a, b^ ^a^Results are corrected for exclusion of economically inactive.^b^ Austria and Switzerland not shown due to differences in the classification.

Relative inequalities in occupational class mortality followed a social gradient with increased risk of death for lower classes, for almost all causes of death studied. However, the magnitude of these inequalities varied considerably between populations. All-cause mortality rate ratios comparing unskilled manual with upper non-manual workers ranged from 1.72 (95% CI: 1.35–2.20) in Tuscany to 3.47 (3.25–3.70) in Denmark. Large relative inequalities in all-cause mortality were also found in Finland. Farmers and self-employed generally showed low mortality rate ratios. An exception, however, is Lithuania where rate ratios for this group were even higher than for the unskilled manual workers (Figure 1).

Generally, occupational class differences were less pronounced for cancer mortality compared to other causes of death, with rate ratios for unskilled manual workers ranging between 1.18 (0.90–1.54) in England and Wales and 2.30 (1.81–2.92) in France. Relative inequalities in CVD mortality were largest in Finland (3.46 (3.37–3.64)), and smallest in Southern European populations, especially in Turin (1.45 (1.05–2.02)) and Tuscany (1.43 (0.83–2.45).

Because data from Switzerland and Austria did not allow a distinction in five occupational classes, we also compared combined groups of non-manual and manual workers (Appendix S3). In this analysis, Switzerland was among the populations with the largest relative occupational class inequalities in all-cause mortality, together with Finland, Denmark, and Lithuania. Inequalities were smaller in Austria, with values closer to England and Wales. Switzerland had relatively large occupational class inequalities in cancer mortality; Austria had relatively small occupational class inequalities in cancer and CVD mortality.

Similar international patterns were seen when we looked at absolute inequalities in mortality, as measured by rate differences between the highest and the lowest occupational classes (Table 3). For all-cause mortality these were largest in Finland, Denmark, France, and Lithuania and smallest in the Netherlands, Madrid, and Tuscany. The main causes contributing to larger absolute inequalities in all-cause mortality differed strongly between countries: in both Finland and Lithuania external causes made the largest contribution, while cancer and all other causes were the most important contributors in Denmark and France, respectively.

**Table 3:** Rate difference (RD)^a^ of all-cause and cause-specific mortality between occupational classes, men, age 30–59^b^.

	All causes	All cancer	All CVD	All external	All other
NORTH					
**Finland**	458	53	126	151	129
**Sweden**	225	28	49	41	50
**Denmark**	369	69	61	58	133
WEST					
**England & Wales**	197	15	76	34	75
**Scotland**	252	51	62	50	76
**Netherlands**	157	44	52	27	27
**France**	381	135	48	72	116
**Switzerland** c **^,^** d **^,^**	158	46	28	30	42
**Austria** c	201	84	46	13	53
SOUTH					
**Spain (Basque)**	228	82	40	35	72
**Spain (Madrid)**	161	71	6	9	78
**Italy (Turin)**	189	77	18	16	84
**Italy (Tuscany)**	110	36	15	15	44
BALTIC					
**Lithuania**	509	83	135	201	75

a RD calculated comparing ASMR (deaths per 100,000 person-years) of unskilled manual and upper non-manual workers.

b Results are corrected for exclusion of economically inactive.

c Both non-manual and manual categories combined.

d Both non-manual categories combined.

Table 4 shows the PAF, calculated including farmers and self-employed. The largest PAFs for all-cause mortality were found in Finland, Denmark, and Lithuania, where 51–57% of all deaths could be saved if all occupational classes experienced the same mortality rates as upper non-manual workers. The smallest PAFs for all-cause mortality were found in the Netherlands and Tuscany.

**Table 4:** Population attributable fraction (PAF)^a^ (in %) of lower occupational class for all-cause and cause-specific mortality, men, age 30–59^b^.

	All causes	All cancer	All CVD	All external	All other
NORTH					
**Finland**	51	33	51	54	61
**Sweden**	42	23	40	43	62
**Denmark**	53	31	48	61	64
WEST					
**England & Wales**	28	7	34	32	51
**Scotland**	41	23	28	66	55
**Netherlands**	22	11	31	24	29
**France**	38	29	41	38	49
**Switzerland** c **^,^** d	(12)	(3)	(10)	(30)	(0)
**Austria** c	(18)	(10)	(12)	(23)	(28)
SOUTH					
**Spain (Basque)**	41	31	39	53	55
**Spain (Madrid)**	33	23	30	39	46
**Italy (Turin)**	34	32	21	21	62
**Italy (Tuscany)**	21	18	11	18	41
BALTIC					
**Lithuania**	57	46	49	63	72

a Reference category are upper non-manual workers.

b Results are corrected for the exclusion of economically inactive.

c Both non-manual and manual categories combined.

d Both non-manual categories combined.

## Discussion

### Summary of findings

Our study is the first to document occupational class differences in all-cause and cause-specific mortality in Europe in the early 2000s, and covers an exceptionally large number of countries. We found that inequalities in mortality by occupational class are still present, and follow a social gradient for almost all populations and causes of death studied. The magnitude of occupational class differences in mortality, however, varied substantially between populations. Relative and absolute inequalities in all-cause mortality were more pronounced in Finland, Denmark, France, and Lithuania than in other populations, and the same countries (except France) also had the highest PAF values for all-cause mortality.

### Methodological issues

In cross-country comparisons, comparability between data is crucial. Occupational class was therefore carefully harmonised between populations, using the well-known EGP scheme [19]. However, we cannot rule out that in some populations the occupational class structure does not entirely fit into this scheme, and misclassifications may lead to over- or under-estimation of inequalities in mortality. A sensitivity analysis with a classification by occupational class which is less sensitive to measurement error (comparing non-manual with manual workers, see Appendix S3) showed roughly the same pattern of mortality inequalities. The same sensitivity analysis showed that Switzerland and Austria, in which non-manual workers could not be subdivided, had large and moderate relative inequalities in all-cause mortality, respectively (see Appendix S3).

Although skilled manual and lower non-manual classes may to some extent reflect the same position in the labour market, we found that the mortality was generally higher in the first than in the second group, except in France where lower non-manual workers had a (non-significantly) higher mortality risk than skilled manual workers. This pattern has been documented for a long time [25] and is explained by a high prevalence of risk factors among lower non-manual workers [26] combined with a deterioration of their status within the population since the 1980s [27].

The combined group of farmers and self-employed is very heterogeneous. Farmers are a very small group in all populations except Lithuania, where farmers and farm labourers account for 21.3% of the population and have the worst health [28]. This may explain why, when analyses are restricted to non-manual and manual workers, Lithuania does not have much larger relative inequalities in mortality than other populations, whereas it has extremely large educational differences in mortality [7]. Lithuania is a special case because mortality inequalities operate at substantially higher absolute levels of mortality. Even the group of upper non-manual employees, who have the lowest mortality in Lithuania, have higher mortality than unskilled manual workers in many other countries.

The smaller inequalities observed in Southern European populations might partly be explained by the specific settings of our populations. These covered mostly urban and more prosperous areas that are not necessarily representative of the whole country in terms of occupational class distributions. Indeed, compared to other European populations in our study, the Southern European populations have smaller proportions of unskilled manual workers (Table 1). However, this does not necessarily imply that estimates of inequalities in mortality between occupational classes are biased. Smaller inequalities in all-cause mortality in the Southern European populations than in other European countries were also found using national data from both Spain [29] and Italy [30].

Cross-country comparisons restricted to active men are likely to lead to biased conclusions for two main reasons. First, inequalities are underestimated when economically inactive men are excluded. Second, the magnitude of this underestimation varies strongly between populations as shown in Appendix S4. We applied an algorithm to account for the exclusion of economically inactive men and carefully assessed the quality of this adjustment. This algorithm provided accurate estimates of occupational class differences in mortality among the whole population (active and inactive) for the four populations studied (Appendix S2). Any remaining bias is likely to be modest.

A more general issue when studying cause-specific mortality is misclassification of causes of death [8]. This, however, should not be a substantial problem in this study because misclassification is most likely to happen within, and not between, the broad categories of causes of death analysed here. Causes of death would have to vary systematically by occupational class and country to affect our results.

### Comparison with previous studies

Although not directly comparable, our results from the early 2000s resemble those from previous studies of occupational class differences in mortality in Europe in several respects [5], [8], [31]. In particular the general geographical pattern of larger inequalities in Northern and Western compared to Southern Europe seems to have been stable over time. However, trends in mortality inequalities differed substantially between countries. Whereas inequalities in mortality by occupational class in Southern Europe did not change a lot over time, they did change in some other countries, e.g. Denmark where differences in total and CVD mortality seem to have increased drastically [8]. A widening of occupational class inequalities in mortality could to some extent be due to the fact that the proportion of the population in lower occupational classes has generally decreased, and that these groups have probably become more selected in terms of e.g., material circumstances or health behaviour [3]–[5]. If this development has taken place earlier in Northern compared to Southern Europe, it could also partly explain the smaller inequalities in Southern Europe.

Educational inequalities in mortality for the early 2000s have recently been analysed by Lundberg et al. [18]. When focusing on the age-category 30–59, we found that relative inequalities in mortality were similar for educational and occupational class in all countries, except in Finland and Denmark, where inequalities were larger for occupational class (results not shown). The latter finding may be due to chance as a study directly comparing occupational and educational differences [32], for an earlier time-period though, found that educational differences were larger than occupational mortality differences in Finland.

### Interpretation of the results

Although the Northern European countries are characterized by a high level of welfare and social security and relatively low levels of income inequalities, many studies including this one have found that inequalities in mortality are not smaller than in other European countries [5], [7], [8], [22], [33]–[35]. To the contrary, we even found comparatively large differences in some of the Nordic countries, i.e. in Finland and Denmark, but not in Sweden. These differences between the Nordic countries may reflect differences in the social patterning of behavioural risk factors [20], or differences in welfare state and labour market programmes and regulations that generate larger inequalities in mortality in some countries than in others [36]. Although all Nordic countries have universal welfare policies, strategies differ when it comes to specific social protection, labour market, taxation, or educational policies [36].

In contrast to education, which is usually fixed early in adulthood, occupational class may change during the whole working life, and thus indicates one's social class closer to death. On the other hand, occupational class inequalities in health are also more sensitive than educational inequalities to direct health selection, because good and bad health influences the likelihood of moving between occupational classes [37], [38]. Differences between countries in the extent of occupational class mobility, and in the extent of health-related selection during occupational class mobility, may therefore partly explain our findings. For example, one could hypothesize that larger occupational class inequalities in Finland, Denmark, France, and/or Lithuania are related to larger health-related selection during social mobility. More research is, however, necessary to investigate these and other hypotheses.

Specific occupational health risks are generally more prevalent in lower occupational classes, and are likely to explain part of the association between occupational class and mortality [39]. These risk factors include physical risk factors such as exposure to noise and pollution, heavy lifting, and risk of injuries, as well as psychosocial risk factors like low prestige, demand and control imbalance, and income deprivation [14], [40]–[42]. Psychosocial factors have been associated with inequalities in CVD mortality [43], [44] whereas exposure to hazards in industries has been shown to explain some of the occupational class differences in cancer mortality [45]–[48].

Socioeconomic inequalities in health care use may also account for part of the occupational class differences in mortality. Lesser availability of treatments and prevention measures among people from manual classes were found to partly explain the slower decrease in CVD mortality as compared to non-manual classes [49]. Inequalities in mortality from conditions amenable to health care were found to explain a substantial part of mortality inequalities in Central and Eastern Europe [7], [50], and inequalities in access to health care could thus partly account for the inequalities in mortality in Lithuania.

Health behaviour is one of the most frequently discussed explanatory factors for the association between occupational class and mortality [51], [52]. Not only during adolescence, but also in adulthood healthy behaviours may be encouraged or discouraged by the social environment, and health-related behaviours may therefore be as strongly or even more strongly related to occupational class as it is to education [3], [53]. Smoking could be one explanation for differences in occupational class inequalities – not least for CVD mortality – between Northern and Southern European populations [54]. While in Northern Europe smoking has declined in higher socioeconomic classes, leading to an increase in relative inequalities in smoking [55], in Southern Europe relatively high rates of smoking are still found in higher socioeconomic classes [53]. Differences in excessive alcohol consumption may also explain part of the occupational class inequalities in cancer and CVD mortality. This could be especially true in countries such as Finland, Denmark, France, Scotland, and Lithuania where high alcohol consumption rates have been reported [56], [57] and higher levels of binge drinking were found in lower socioeconomic groups [58]. This may also partly explain the large inequalities in mortality from external causes in some of these populations.

## Conclusions

Our study not only provides the most recent, but also the most comprehensive comparative analysis of occupational class inequalities in mortality in Europe. It shows that these inequalities have persisted into the 21^st^ century, are present in all countries with available data, and may even have widened on a relative scale in some countries. Furthermore, our analysis of PAF showed that, despite diminishing shares of lower occupational classes in the total population, the impact of occupational class inequalities on population health is still very substantial. The fact that the average mortality rate in the population as a whole would decrease by between a quarter and a half, if the mortality rates of lower occupational classes would be reduced to those of the upper non-manual class, should be a stimulus to policy-makers to start taking health inequalities seriously.

## Supporting Information

Appendix S1 **Correction for the exclusion of economically inactive men.**
(DOCX)

Appendix S2 **Performance of the correction used to account for the exclusion of economically active men.**
(DOCX)

Appendix S3 **Mortality of manual workers compared to non-manual workers among men in 14 populations.**
(DOCX)

Appendix S4 **Mortality rate ratios by occupational class among economically active men in 14 populations.**
(DOCX)
