# Supplementary material for: Occupational Class Inequalities in All-Cause and Cause-Specific Mortality among Middle-Aged Men in 14 European Populations during the Early 2000s
Source: PLoS One. 2014 Sep 30;9(9):e108072. doi: 10.1371/journal.pone.0108072 (PMC4182439; doi:10.1371/journal.pone.0108072)
Supplement: Appendix S1 — Correction for the exclusion of economically inactive men. (DOCX) [file pone.0108072.s001.docx]

**Appendix S1 – Correction for the exclusion of economically inactive men**

We applied the algorithm developed by [Kunst and Groenhof (1996](#_ENREF_23)) to account for the exclusion of inactive men when computing occupational class differences in mortality.

A correction formula was derived to estimate the mortality differences by occupational class taking into account economically inactive men. The idea is to compute a correction factor that represents the extent to which the mortality rate among active men in each occupational class should be increased, in order to obtain an estimate of the mortality rate among all men in this occupational class.

For the occupational class z, the formula is:

$1+P_{z}^{inactive}*(RR_{z}^{inactive/active}-1)$ (1)

with $P_{z}^{inactive}$ being the proportion of inactive men by occupational class, and $RR_{z}^{inactive/active}$ being the mortality rate ratio of inactive compared to active men by occupational class.

For each of these two input information, an assumption was made.

Regarding the proportion of economically inactive men by occupational class, it was assumed that for each occupational class, this proportion was proportional to the share of inactive men in the whole population. We also assumed that *this relative difference differed by occupational class, but did not differ between countries*. It can be written as follows:

$$P_{z}^{inactive}=const_{z}*P_{all}^{inactive}$$

The const_z_ corresponds to the proportion of inactive men in class z when compared with the whole population. It was computed from National Health Interview Survey data.

Regarding the mortality rate ratio of inactive compared to active men by occupational class, it was assumed that *this rate ratio was constant across occupational groups and equalled that computed among the total population*. This rate ratio was derived from the mortality dataset.

These assumptions were checked in the available mortality and morbidity data (see Appendix 2).

With these two assumptions, the formula (1) can be written as:

$1+P_{z}^{inactive}*(RR_{z}^{inactive/active}-1)$ (2)

Formula (2) can be extended to a formula for the comparison of any occupational class, say, a lower class z and a higher class y.

$\frac{1+const_{z}*P_{all}^{inactive}*(RR_{all}^{inactive/active}-1)}{1+const_{y}*P_{all}^{inactive}*(RR_{all}^{inactive/active}-1)}$ (3)

Formula (3) was then applied to the rate ratios estimated excluding economically inactive men.

Table A1.1 and A1.2 show the input information we needed to calculate the correction factor to adjust for the exclusion of economically inactive men.

Table S1.1: Proportion of inactive in the total population and mortality rate ratios of

inactive vs. active, men, age 30-59.

|  | **Proportion of inactive in total population** | **Mortality RR of the inactive vs. active men** | | | | |
| --- | --- | --- | --- | --- | --- | --- |
|  |  | **All causes** | **All cancer** | **All CVD** | **All external** | **All other** |
| NORTH | | | | | | |
| **Finland** | 12% | 3.33 | 2.33 | 3.45 | 2.84 | 4.85 |
| **Sweden** | 17% | 4.25 | 2.06 | 3.84 | 4.32 | 8.64 |
| **Denmark** | 12% | 6.36 | 2.61 | 4.31 | 5.45 | 11.85 |
| WEST | | | | | | |
| **England** | 13% | 3.84 | 2.71 | 3.32 | 3.60 | 7.35 |
| **Scotland** | 15% | 4.30 | 2.09 | 3.79 | 3.05 | 9.66 |
| **Netherlands** | 10% | 3.45 | 2.71 | 2.84 | 2.74 | 6.82 |
| **France** | 16% | 3.97 | 2.77 | 4.03 | 2.68 | 7.74 |
| **Switzerland** | 9% | 4.62 | 2.75 | 3.21 | 2.89 | 9.60 |
| **Austria** | 14% | 5.06 | 4.69 | 3.77 | 2.90 | 12.55 |
| SOUTH | | | | | | |
| **Spain (Basque)** | 9% | 3.50 | 2.76 | 2.96 | 2.86 | 7.04 |
| **Spain (Madrid)** | 15% | 3.64 | 2.96 | 2.80 | 2.61 | 5.83 |
| **Italy (Turin)** | 17% | 2.81 | 2.28 | 1.97 | 2.10 | 6.84 |
| **Italy (Tuscany)** | 15% | 3.03 | 2.07 | 2.74 | 2.13 | 7.44 |
| BALTIC | | | | | | |
| **Lithuania** | 29% | 2.89 | 2.30 | 2.89 | 2.46 | 4.97 |

Source: Mortality data

Table S1.2: Relative difference of the proportion of economically inactive men by

occupational class when compared to the proportion of inactive men in total

population, derived from National Health Interview Survey data.

| **Population** | **Upper non-manual** | **Lower non-manual** | **Skilled manual** | **Unskilled manual** |
| --- | --- | --- | --- | --- |
| **All countries except Switzerland and Austria** | 0.6 | 1 | 1.3 | 1.7 |
| **Switzerland** | 0.8 | - | 1.5 | - |
| **Austria** | 0.8 | - | 1.3 | 1.7 |
