# Supplementary material for: Occupational Class Inequalities in All-Cause and Cause-Specific Mortality among Middle-Aged Men in 14 European Populations during the Early 2000s
Source: PLoS One. 2014 Sep 30;9(9):e108072. doi: 10.1371/journal.pone.0108072 (PMC4182439; doi:10.1371/journal.pone.0108072)
Supplement: Appendix S2 — Performance of the correction used to account for the exclusion of economically active men. (DOCX) [file pone.0108072.s002.docx]

**Appendix S2 – Performance of the correction used to account for the exclusion of economically active men**

Table A2.1 illustrates the performance of the algorithm correcting for the exclusion of inactive men. Four populations are presented, where occupational class was available for all men (active and inactive): Finland, England & Wales, Basque Country and Turin.

For each population, the first column shows the rate ratios by occupational class calculated without inactive men. The second and third columns show the corrected rate ratios (estimated when using the algorithm) using two different corrections. For each population, we used two sets of values for the proportion of economically inactive men by occupational class when compared with the total population: in column 2 the values come from the mortality data and in column 3 from the National Health Interview Survey. The fourth column shows the rate ratios by occupational class computed among the whole population including economically inactive persons.

When comparing columns one and four we see that excluding economically inactive men leads to an underestimation of mortality. The comparison of column two or three and four shows that the developed correction algorithm seems to work well and adequately corrects for the exclusion of inactive men.

The results presented in this paper are corrected as shown in column three (Corr. 2).

Table S2.1: Rate ratio of all-cause and cause-specific mortality in 4 countries; using 4 different methods, men, age 30-59.

|  | **Finland** | | | | **England & Wales** | | | | **Basque Country** | | | | **Turin** | | | |
| --- | --- | --- | --- | --- | --- | --- | --- | --- | --- | --- | --- | --- | --- | --- | --- | --- |
|  | without inactive | Corr. 1 | Corr. 2 | with inactive | without inactive | Corr. 1 | Corr. 2 | with inactive | without inactive | Corr. 1 | Corr. 2 | with inactive | without inactive | Corr. 1 | Corr. 2 | with inactive |
| **All causes** |  |  |  |  |  |  |  |  |  |  |  |  |  |  |  |  |
| Upper non-manual | 1 | 1 | 1 | 1 | 1 | 1 | 1 | 1 | 1 | 1 | 1 | 1 | 1 | 1 | 1 | 1 |
| Lower non-manual | 1.43 | 1.50 | 1.57 | 1.50 | 0.94 | 1.02 | 1.05 | 1.13 | 1.41 | 1.57 | 1.53 | 1.46 | 1.20 | 1.40 | 1.33 | 1.35 |
| Skilled manual | 2.06 | 2.21 | 2.40 | 2.20 | 1.31 | 1.50 | 1.59 | 1.58 | 1.62 | 1.84 | 1.86 | 1.69 | 1.44 | 1.81 | 1.71 | 1.68 |
| Unskilled manual | 2.73 | 3.13 | 3.42 | 2.89 | 1.59 | 2.04 | 2.12 | 1.89 | 1.87 | 2.17 | 2.29 | 1.92 | 1.69 | 2.21 | 2.17 | 2.04 |
| **All cancer** |  |  |  |  |  |  |  |  |  |  |  |  |  |  |  |  |
| Upper non-manual | 1 | 1 | 1 | 1 | 1 | 1 | 1 | 1 | 1 | 1 | 1 | 1 | 1 | 1 | 1 | 1 |
| Lower non-manual | 1.31 | 1.35 | 1.39 | 1.37 | 0.71 | 0.75 | 0.77 | 0.70 | 1.33 | 1.43 | 1.41 | 1.30 | 1.30 | 1.46 | 1.41 | 1.41 |
| Skilled manual | 1.55 | 1.62 | 1.70 | 1.73 | 1.15 | 1.26 | 1.31 | 1.34 | 1.39 | 1.53 | 1.54 | 1.41 | 1.58 | 1.87 | 1.80 | 1.83 |
| Unskilled manual | 1.81 | 1.98 | 2.09 | 2.00 | 0.97 | 1.15 | 1.18 | 1.22 | 1.60 | 1.78 | 1.86 | 1.56 | 1.69 | 2.07 | 2.05 | 1.98 |
| **All CVD** |  |  |  |  |  |  |  |  |  |  |  |  |  |  |  |  |
| Upper non-manual | 1 | 1 | 1 | 1 | 1 | 1 | 1 | 1 | 1 | 1 | 1 | 1 | 1 | 1 | 1 | 1 |
| Lower non-manual | 1.49 | 1.56 | 1.64 | 1.59 | 1.01 | 1.09 | 1.12 | 1.28 | 1.49 | 1.63 | 1.60 | 1.55 | 1.07 | 1.17 | 1.14 | 1.09 |
| Skilled manual | 2.00 | 2.15 | 2.34 | 2.24 | 1.51 | 1.69 | 1.78 | 1.81 | 1.60 | 1.78 | 1.79 | 1.62 | 1.28 | 1.47 | 1.42 | 1.29 |
| Unskilled manual | 2.74 | 3.17 | 3.46 | 3.05 | 1.96 | 2.43 | 2.51 | 2.18 | 1.74 | 1.97 | 2.06 | 1.76 | 1.25 | 1.47 | 1.45 | 1.41 |
| **All external** |  |  |  |  |  |  |  |  |  |  |  |  |  |  |  |  |
| Upper non-manual | 1 | 1 | 1 | 1 | 1 | 1 | 1 | 1 | 1 | 1 | 1 | 1 | 1 | 1 | 1 | 1 |
| Lower non-manual | 1.37 | 1.42 | 1.47 | 1.41 | 0.53 | 0.57 | 0.59 | 0.66 | 1.57 | 1.70 | 1.67 | 1.65 | 0.88 | 0.97 | 0.94 | 0.89 |
| Skilled manual | 2.35 | 2.49 | 2.66 | 2.35 | 1.01 | 1.14 | 1.21 | 1.05 | 2.37 | 2.62 | 2.64 | 2.62 | 1.01 | 1.17 | 1.13 | 1.09 |
| Unskilled manual | 3.14 | 3.52 | 3.78 | 3.20 | 1.87 | 2.36 | 2.44 | 1.97 | 2.34 | 2.63 | 2.74 | 2.44 | 1.41 | 1.68 | 1.67 | 1.51 |
| **All other** |  |  |  |  |  |  |  |  |  |  |  |  |  |  |  |  |
| Upper non-manual | 1 | 1 | 1 | 1 | 1 | 1 | 1 | 1 | 1 | 1 | 1 | 1 | 1 | 1 | 1 | 1 |
| Lower non-manual | 1.63 | 1.75 | 1.87 | 1.64 | 1.88 | 2.17 | 2.31 | 2.25 | 1.45 | 1.79 | 1.71 | 1.71 | 1.58 | 2.25 | 2.00 | 2.41 |
| Skilled manual | 2.47 | 2.74 | 3.09 | 2.49 | 1.69 | 2.13 | 2.36 | 2.15 | 1.83 | 2.37 | 2.39 | 2.10 | 1.92 | 3.18 | 2.78 | 2.84 |
| Unskilled manual | 3.43 | 4.21 | 4.75 | 3.31 | 2.58 | 3.89 | 4.13 | 2.95 | 2.60 | 3.49 | 3.82 | 2.93 | 3.06 | 5.49 | 5.16 | 4.55 |

Correction 1: Calculated using the relative difference of the proportion of economically inactive men by occupational class, when compared to the proportion of inactive men in total population, derived from the country specific mortality data.

Correction 2: Calculated using the relative difference of the proportion of economically inactive men by occupational class, when compared to the proportion of inactive men in total population, derived from National Health Interview Survey data.
