# Supplementary material for: Occupational Class Inequalities in All-Cause and Cause-Specific Mortality among Middle-Aged Men in 14 European Populations during the Early 2000s
Source: PLoS One. 2014 Sep 30;9(9):e108072. doi: 10.1371/journal.pone.0108072 (PMC4182439; doi:10.1371/journal.pone.0108072)
Supplement: Appendix S3 — Mortality of manual workers compared to non-manual workers among men in 14 populations. (DOCX) [file pone.0108072.s003.docx]

**Appendix S3 – Mortality of manual workers compared to non-manual workers among men in 14 populations**

Table S3.1: Age-standardised rate (ASMR)^a^ and rate ratio (RR) of all-cause and cause-specific mortality, comparing manual workers to non-manual workers, corrected for the exclusion of economically inactive, men, age 30-59.

|  | **All causes** | | | **All cancer** | | | **All CVD** | | | **All external** | | | **All other** | | |
| --- | --- | --- | --- | --- | --- | --- | --- | --- | --- | --- | --- | --- | --- | --- | --- |
|  | ASMR | RR | (95% CI) | ASMR | RR | (95% CI) | ASMR | RR | (95% CI) | ASMR | RR | (95% CI) | ASMR | RR | (95% CI) |
| NORTH | | | | | | | | | | | | | | | |
| **Finland** |  |  |  |  |  |  |  |  |  |  |  |  |  |  |  |
| Non-manual | 229 | 1 |  | 57 | 1 |  | 63 | 1 |  | 64 | 1 |  | 45 | 1 |  |
| Manual | 510 | 1.99 | (1.93-2.04) | 89 | 1.55 | (1.45-1.64) | 136 | 1.98 | (1.88-2.09) | 162 | 2.25 | (2.13-2.38) | 121 | 2.13 | (2.01-2.26) |
| **Sweden** |  |  |  |  |  |  |  |  |  |  |  |  |  |  |  |
| Non-manual | 161 | 1 |  | 53 | 1 |  | 37 | 1 |  | 31 | 1 |  | 18 | 1 |  |
| Manual | 306 | 1.9 | (1.84-1.97) | 72 | 1.38 | (1.30-1.46) | 66 | 1.78 | (1.67-1.90) | 61 | 1.99 | (1.85-2.15) | 48 | 2.65 | (2.42-2.90) |
| **Denmark** |  |  |  |  |  |  |  |  |  |  |  |  |  |  |  |
| Non-manual | 172 | 1 |  | 71 | 1 |  | 38 | 1 |  | 22 | 1 |  | 36 | 1 |  |
| Manual | 390 | 2.37 | (2.26-2.48) | 116 | 1.62 | (1.51-1.74) | 75 | 1.96 | (1.79-2.16) | 60 | 2.69 | (2.39-3.03) | 110 | 3.07 | (2.80-3.37) |
| WEST | | | | | | | | | | | | | | | |
| **England & Wales** |  |  |  |  |  |  |  |  |  |  |  |  |  |  |  |
| Non-manual | 173 | 1 |  | 75 | 1 |  | 49 | 1 |  | 22 | 1 |  | 26 | 1 |  |
| Manual | 312 | 1.76 | (1.57-1.96) | 91 | 1.31 | (1.08-1.57) | 105 | 1.99 | (1.63-2.44) | 43 | 1.71 | (1.23-2.36) | 73 | 2.34 | (1.85-2.95) |
| **Scotland** |  |  |  |  |  |  |  |  |  |  |  |  |  |  |  |
| Non-manual | 149 | 1 |  | 60 | 1 |  | 42 | 1 |  | 18 | 1 |  | 28 | 1 |  |
| Manual | 309 | 2.05 | (1.68-2.50) | 93 | 1.54 | (1.12-2.13) | 82 | 2 | (1.31-2.78) | 52 | 2.59 | (1.53-4.38) | 75 | 2.75 | (1.77-4.27) |
| **Netherlands** |  |  |  |  |  |  |  |  |  |  |  |  |  |  |  |
| Non-manual | 167 | 1 |  | 75 | 1 |  | 45 | 1 |  | 21 | 1 |  | 26 | 1 |  |
| Manual | 296 | 1.62 | (1.42-1.84) | 105 | 1.31 | (1.07-1.60) | 93 | 1.82 | (1.44-2.30) | 41 | 2.15 | (1.52-3.04) | 55 | 1.87 | (1.37-2.57) |
| **France** |  |  |  |  |  |  |  |  |  |  |  |  |  |  |  |
| Non-manual | 250 | 1 |  | 109 | 1 |  | 35 | 1 |  | 53 | 1 |  | 53 | 1 |  |
| Manual | 465 | 1.83 | (1.64-2.04) | 188 | 1.67 | (1.41-1.98) | 74 | 2.22 | (1.66-2.97) | 86 | 1.61 | (1.27-2.04) | 110 | 2.02 | (1.59-2.57) |
| **Switzerland** |  |  |  |  |  |  |  |  |  |  |  |  |  |  |  |
| Non-manual | 162 | 1 |  | 59 | 1 |  | 36 | 1 |  | 35 | 1 |  | 32 | 1 |  |
| Manual | 322 | 2,17 | (2.06-2.28) | 104 | 1,76 | (1.61-1.93) | 64 | 1,77 | (1.58-1.98) | 65 | 1,86 | (1.67-2.08) | 75 | 2,36 | (2.10-2.65) |
| **Austria** |  |  |  |  |  |  |  |  |  |  |  |  |  |  |  |
| Non-manual | 199 | 1 |  | 67 | 1 |  | 60 | 1 |  | 48 | 1 |  | 24 | 1 |  |
| Manual | 343 | 1.73 | (1.60-1.88) | 103 | 1.49 | (1.29-1.73) | 91 | 1.5 | (1.29-1.74) | 83 | 1.75 | (1.51-2.02) | 60 | 2.6 | (2.10-3.22) |

| SOUTH | | | | | | | | | | | | | | | |
| --- | --- | --- | --- | --- | --- | --- | --- | --- | --- | --- | --- | --- | --- | --- | --- |
| **Spain (Basque)** |  |  |  |  |  |  |  |  |  |  |  |  |  |  |  |
| Non-manual | 213 | 1 |  | 110 | 1 |  | 46 | 1 |  | 25 | 1 |  | 32 | 1 |  |
| Manual | 350 | 1.48 | (1.58-1.78) | 156 | 1.3 | (1.32-1.56) | 70 | 1.39 | (1.36-1.75) | 52 | 1.94 | (1.83-2.51) | 70 | 1.8 | (1.98-2.65) |
| **Spain (Madrid)** |  |  |  |  |  |  |  |  |  |  |  |  |  |  |  |
| Non-manual | 236 | 1 |  | 102 | 1 |  | 49 | 1 |  | 23 | 1 |  | 61 | 1 |  |
| Manual | 348 | 1.48 | (1.39-1.58) | 150 | 1.46 | (1.32-1.61) | 56 | 1.16 | (0.99-1.35) | 38 | 1.62 | (1.34-1.95) | 102 | 1.69 | (1.49-1.91) |
| **Italy (Turin)** |  |  |  |  |  |  |  |  |  |  |  |  |  |  |  |
| Non-manual | 171 | 1 |  | 76 | 1 |  | 48 | 1 |  | 22 | 1 |  | 24 | 1 |  |
| Manual | 289 | 1.6 | (1.45-1.76) | 127 | 1.6 | (1.38-1.86) | 64 | 1.29 | (1.06-1.58) | 32 | 1.37 | (1.01-1.86) | 66 | 2.23 | (1.77-2.81) |
| **Italy (Tuscany)** |  |  |  |  |  |  |  |  |  |  |  |  |  |  |  |
| Non-manual | 161 | 1 |  | 77 | 1 |  | 36 | 1 |  | 21 | 1 |  | 27 | 1 |  |
| Manual | 235 | 1.48 | (1.27-1.72) | 99 | 1.28 | (1.02-1.60) | 45 | 1.41 | (1.01-1.97) | 35 | 1.91 | (1.29-2.84) | 55 | 2.24 | (1.58-3.18) |
| BALTIC | | | | | | | | | | | | | | | |
| **Lithuania** |  |  |  |  |  |  |  |  |  |  |  |  |  |  |  |
| Non-manual | 327 | 1 |  | 78 | 1 |  | 113 | 1 |  | 105 | 1 |  | 31 | 1 |  |
| Manual | 708 | 2.16 | (2.04-2.29) | 143 | 1.78 | (1.58-2.01) | 214 | 1.89 | (1.71-2.08) | 257 | 2.41 | (2.20-2.65) | 86 | 2.82 | (2.36-3.37) |

^a^ Direct age standardised mortality rates.
