# Supplementary material for: Occupational Class Inequalities in All-Cause and Cause-Specific Mortality among Middle-Aged Men in 14 European Populations during the Early 2000s
Source: PLoS One. 2014 Sep 30;9(9):e108072. doi: 10.1371/journal.pone.0108072 (PMC4182439; doi:10.1371/journal.pone.0108072)
Supplement: Appendix S4 — Mortality rate ratios by occupational class among economically active men in 14 populations. (DOCX) [file pone.0108072.s004.docx]

**Appendix S4 – Mortality rate ratios by occupational class among economically active men in 14 populations**

Table S4.1: Rate ratio of all-cause and cause-specific mortality among economically active men,

age 30-59.

|  | **All causes** | **All cancer** | **All CVD** | **All external** | **All other** |
| --- | --- | --- | --- | --- | --- |
| NORTH | | | | | |
| **Finland** |  |  |  |  |  |
| Upper non-manual | 1 | 1 | 1 | 1 | 1 |
| Lower non-manual | 1.43 | 1.31 | 1.49 | 1.37 | 1.63 |
| Skilled manual | 2.06 | 1.55 | 2 | 2.35 | 2.47 |
| Unskilled manual | 2.73 | 1.81 | 2.74 | 3.14 | 3.43 |
| **Sweden** |  |  |  |  |  |
| Upper non-manual | 1 | 1 | 1 | 1 | 1 |
| Lower non-manual | 1.41 | 1.21 | 1.55 | 1.01 | 2.27 |
| Skilled manual | 1.66 | 1.26 | 1.62 | 1.82 | 2.9 |
| Unskilled manual | 2.34 | 1.8 | 2.43 | 2.47 | 3.83 |
| **Denmark** |  |  |  |  |  |
| Upper non-manual | 1 | 1 | 1 | 1 | 1 |
| Lower non-manual | 1.21 | 1.08 | 1.4 | 1.37 | 1.15 |
| Skilled manual | 1.85 | 1.42 | 1.73 | 2.28 | 1.79 |
| Unskilled manual | 2.31 | 1.68 | 2.09 | 2.74 | 2.61 |
| WEST | | | | | |
| **England & Wales** | |  |  |  |  |
| Upper non-manual | 1 | 1 | 1 | 1 | 1 |
| Lower non-manual | 0.94 | 0.71 | 1.01 | 0.53 | 1.88 |
| Skilled manual | 1.31 | 1.15 | 1.51 | 1.01 | 1.69 |
| Unskilled manual | 1.59 | 0.97 | 1.96 | 1.87 | 2.58 |
| **Scotland** |  |  |  |  |  |
| Upper non-manual | 1 | 1 | 1 | 1 | 1 |
| Lower non-manual | 1.41 | 1.21 | 1.55 | 1.01 | 2.27 |
| Skilled manual | 1.66 | 1.26 | 1.62 | 1.82 | 2.9 |
| Unskilled manual | 2.34 | 1.8 | 2.43 | 2.47 | 3.83 |
| **Netherlands** |  |  |  |  |  |
| Upper non-manual | 1 | 1 | 1 | 1 | 1 |
| Lower non-manual | 1.22 | 1.1 | 1.6 | 1.17 | 1 |
| Skilled manual | 1.31 | 1.05 | 1.71 | 1.45 | 1.28 |
| Unskilled manual | 1.66 | 1.38 | 1.96 | 2.05 | 1.68 |
| **France** |  |  |  |  |  |
| Upper non-manual | 1 | 1 | 1 | 1 | 1 |
| Lower non-manual | 1.99 | 1.71 | 1.45 | 2.41 | 2.54 |
| Skilled manual | 1.6 | 1.51 | 1.95 | 1.54 | 1.64 |
| Unskilled manual | 1.95 | 1.82 | 1.86 | 2.12 | 2.14 |
| **Switzerland** |  |  |  |  |  |
| Upper non-manual | 1 | 1 | 1 | 1 | 1 |
| Lower non-manual | |  |  |  |  |
| Skilled manual | 1.67 | 1.76 | 1.58 | 1.7 | 1.83 |
| Unskilled manual | |  |  |  |  |
| **Austria** |  |  |  |  |  |
| Upper non-manual | 1 | 1 | 1 | 1 | 1 |
| Lower non-manual | |  |  |  |  |
| Skilled manual | 1.34 | 1.13 | 1.19 | 1.58 | 1.69 |
| Unskilled manual | 1.45 | 1.49 | 1.48 | 1.16 | 1.95 |

| SOUTH | | | | | |
| --- | --- | --- | --- | --- | --- |
| **Spain (Basque)** | |  |  |  |  |
| Upper non-manual | 1 | 1 | 1 | 1 | 1 |
| Lower non-manual | 1.41 | 1.33 | 1.49 | 1.57 | 1.45 |
| Skilled manual | 1.62 | 1.39 | 1.6 | 2.37 | 1.83 |
| Unskilled manual | 1.87 | 1.6 | 1.74 | 2.34 | 2.6 |
| **Spain (Madrid)** | |  |  |  |  |
| Upper non-manual | 1 | 1 | 1 | 1 | 1 |
| Lower non-manual | 1.33 | 1.1 | 1.51 | 1.48 | 1.58 |
| Skilled manual | 1.4 | 1.28 | 1.24 | 1.87 | 1.56 |
| Unskilled manual | 1.71 | 1.54 | 1.47 | 1.58 | 2.29 |
| **Italy (Turin)** |  |  |  |  |  |
| Upper non-manual | 1 | 1 | 1 | 1 | 1 |
| Lower non-manual | 1.2 | 1.3 | 1.07 | 0.88 | 1.58 |
| Skilled manual | 1.44 | 1.58 | 1.28 | 1.01 | 1.92 |
| Unskilled manual | 1.69 | 1.69 | 1.25 | 1.41 | 3.06 |
| **Italy (Tuscany)** | |  |  |  |  |
| Upper non-manual | 1 | 1 | 1 | 1 | 1 |
| Lower non-manual | 1.18 | 1.35 | 0.88 | 0.89 | 1.42 |
| Skilled manual | 1.31 | 1.25 | 1.09 | 1.5 | 1.67 |
| Unskilled manual | 1.34 | 1.28 | 1.14 | 1.38 | 1.78 |
| BALTIC | | | | | |
| **Lithuania** |  |  |  |  |  |
| Upper non-manual | 1 | 1 | 1 | 1 | 1 |
| Lower non-manual | 1.32 | 1.56 | 1.27 | 1.08 | 2.22 |
| Skilled manual | 1.74 | 1.59 | 1.54 | 1.89 | 2.27 |
| Unskilled manual | 1.78 | 1.51 | 1.52 | 2.1 | 2.17 |
